# Evolutionary Aspects of Selenium Binding Protein (SBP)

**DOI:** 10.1007/s00239-023-10105-4

**Published:** 2023-04-11

**Authors:** Irene Dervisi, Chrysanthi Valassakis, Aikaterini Koletti, Vassilis N. Kouvelis, Emmanouil Flemetakis, Christos A. Ouzounis, Andreas Roussis

**Affiliations:** 1 Section of Botany, Department of Biology, National & Kapodistrian University of Athens, 15784 Athens, Greece; 2 Department of Biotechnology, School of Applied Biology and Biotechnology, Agricultural University of Athens, 11855 Athens, Greece; 3 Section of Genetics and Biotechnology, Department of Biology, National & Kapodistrian University of Athens, 15784 Athens, Greece; 4 Biological Computation & Process Laboratory, Centre for Research & Technology Hellas, Chemical Process & Energy Resources Institute, 54124 Thessaloníki, Greece; 5 Biological Computation & Computational Biology Group, AIIA Lab, School of Informatics, Aristotle University of Thessalonica, 57001 Thessaloníki, Greece

**Keywords:** Selenium, SBP, Phylogenetic profiling, Phylogenetic tree, CxxC motif, Motifs

## Abstract

Selenium-binding proteins represent a ubiquitous protein family and recently SBP1 was described as a new stress response regulator in plants. SBP1 has been characterized as a methanethiol oxidase, however its exact role remains unclear. Moreover, in mammals, it is involved in the regulation of anti-carcinogenic growth and progression as well as reduction/oxidation modulation and detoxification. In this work, we delineate the functional potential of certain motifs of SBP in the context of evolutionary relationships. The phylogenetic profiling approach revealed the absence of SBP in the fungi phylum as well as in most non eukaryotic organisms. The phylogenetic tree also indicates the differentiation and evolution of characteristic SBP motifs. Main evolutionary events concern the CSSC motif for which Acidobacteria, Fungi and Archaea carry modifications. Moreover, the CC motif is harbored by some bacteria and remains conserved in Plants, while modified to CxxC in Animals. Thus, the characteristic sequence motifs of SBPs mainly appeared in Archaea and Bacteria and retained in Animals and Plants. Our results demonstrate the emergence of SBP from bacteria and most likely as a methanethiol oxidase.

**Supplementary Information:**

The online version contains supplementary material available at 10.1007/s00239-023-10105-4.

## Introduction

Selenium (Se) was discovered in 1817 by Berzelius yet it was not for another 140 years that the essentiality of this element was broadly realized (Schwarz and Foltz 1957). Selenium is an essential micronutrient for many organisms (Archaea, Bacteria, Protozoa, green algae and Animals) (Birringer et al. 2002). At low doses, selenium can promote growth in plants such as potato (Turakainen et al. 2006), ryegrass (Hartikainen et al. 2000), tea (Hu et al. 2003), rice (Liu et al. 2004) and soybean (Djanaguiraman et al. 2010).

As Sulfur and Selenium both belong to chalcogens they resemble each other chemically. In plants they share similar pathways for uptake and translocation and they are components of proteins as constituents of cysteine, methionine selenocysteine and selenomethionine (Jacob et al. 2003; Hasanuzzaman et al. 2020). However, Selenium is not able to create π bonds and the electrons of its outer valence are looser than those of sulfur (S) (Reich and Hondal 2016) allowing selenium to react with Reactive Oxygen Species (ROS) faster than sulfur. Nonetheless, the inability of creating a π-bond in Se-O allows its easier reduction compared with the respective S–O bonds (Reich and Hondal 2016). Due to this chemical property, Se participates in specific biological processes by replacing sulfur, such as in selenoproteins.

In organisms where selenium is essential, it is required for the biosynthesis of the selenoamino acid Se-Cys (SeC), used for the translation of selenoproteins. Selenoproteins have been identified in several organisms such as mammals, bacteria, archaea and viruses, but not in plants and yeasts (Stadtman 1990). Selenoproteins perform various critical functions in redox reactions, free radical scavenging and hormone regulation (Gladyshev and Kryukov 2001; Kryukov et al. 2003; Driscoll and Copeland 2003; Kryukov and Gladyshev 2004). In land plants, where selenium is not essential, homologues of selenoproteins do not incorporate selenium and therefore have a Cys residue instead of a Se-Cys.

The Selenium Binding Protein (SBP), which does not contain Se-Cys, is most probably involved in selenium metabolism. SBP was initially isolated from mouse liver, as a cytosolic protein and named SBP56 (Bansal et al. 1989, 1990). However, recent studies revealed that SBP56 is a misannotated methanethiol oxidase (Eyice et al. 2018; Pol et al. 2018). The degree of similarity across SBPs is high among mammals (mouse, human) and plants (Arabidopsis) reaching ~ 70%. Moreover, SBP1 has been proposed as a candidate of the selenium delivery system to selenophosphate synthetase (SPS), an enzyme important for selenophosphate production and thus the formation of selenoproteins (Tobe and Mihara 2018).

Human SBP protein (*hs*SBP, SELENBP) plays an important role in key processes such as the regulation of anti-carcinogenic growth and progression, reduction/oxidation modulation, detoxification, intra Golgi protein transport (Chen et al. 2004) and proteasome degradation pathway in a Se-dependent manner. Decreased levels of human SBP1 are associated with various cancers such as ovary, lung, esophagus, colon, stomach, liver and in uterine leiomyoma (Chen et al. 2004; Zhang et al. 2010a, b; Di Stasio et al. 2011; Xia et al. 2011; Huang et al. 2012; Ansong et al. 2015; Udawela et al. 2015). Moreover, *hs*SBP1 has been associated with Behcet's disease and schizophrenia and proposed as a serological marker in immunoscreening methods (Chen et al. 2004) and a biomarker for neurological diseases associated with schizophrenia (Chen et al. 2004; Kanazawa et al. 2009; Amar et al. 2010).

Recent studies have shown that in *Arabidopsis* SBP1 participates in a protein interaction network consisting at least of SBP, a NADP-dependent glyceraldehyde-3-phosphate dehydrogenase (GAPDH) and a fructose-bisphosphate aldolase (FBA) (Agalou et al. 2006). Moreover, it has been shown *in planta* that SBP1 also interacts with glutaredoxins (GRXS14, GRXS16) (Valassakis et al. 2019), a phospholipase (DALL3) (Dervisi et al. 2020) and a papain-like protease (RD19c) (Dervisi et al. 2022). A major step towards understanding SBP1 function in plants and its involvement in selenium metabolism and detoxification mechanisms was the identification of the Se-binding site and the involvement of two Cys residues in *At*SBP1, as well as the function of selenite reduction (Schild et al. 2014).

The bacterial SBP56 is a Cu-dependent methanethiol oxidase (MTO), a widely distributed protein (Eyice et al. 2018). MTOs degrade methanethiol to formaldehyde, hydrogen sulfide (H_2_S) and hydrogen peroxide (H_2_O_2_), an enzyme activity not found in humans, where H_2_S and H_2_O_2_ are known cellular signal molecules. Low levels of SELENBP1 and subsequently reduced MTO activity may cause malodor syndrome and other diseases (Pol et al. 2018).

We have recently shown that in *Chlamydomonas reinhardtii* lack of the SBP1 (SBD1) homologue affects the molecular and biochemical responses upon oxidative stress dramatically. Moreover, interactions of SBP1 with certain proteins in *Arabidopsis* are also conserved in *C. reinhardtii*. The same study revealed that SBP1 is involved in redox early sensing and triggering of the subsequent cellular responses via protein–protein interactions (Koletti et al. 2022).

The diversity of SBPs in many different organisms with differential utilization requires a deeper examination of the evolutionary history of this protein family. The aim of the present study is to understand the functional potential of certain motifs in protein coding sequence of *At*SBP1 among different taxa. We investigate the involvement of these motifs with the function of SBP, in the context of evolutionary relationships, towards a better understanding of their potential roles. In addition, we report a phylogenetic analysis for 120 species (from archaea to mammals) to obtain valuable insights into the evolution of SBP.

## Material and Methods

### Data Sources and Searches

The Reference protein database of NCBI (refseq_protein) was used to retrieve all sequences analyzed in this study. Their selection was based on similarity levels (> 80% query cover and 70% identity) with the *Arabidopsis thaliana* Selenium Binding Protein 1 (UniProt Acc. No. O23264), using NCBI BLAST with default parameters (Johnson et al. 2008; Boratyn et al. 2013). In total, 129 different SBP proteins identified as homologues to *Arabidopsis thaliana* SBP belonging to representative organisms from all Domains of Life were analyzed in detail (Table S1).

For phylogenetic profiling, the latest version (2022_01) of reference proteome collection (excluding viruses) was used (Chen et al. 2011; The UniProt Consortium 2019), comprising 10,273 individual proteomes and 59,653,876 protein sequences.

### Domain Identification

The structure of all Selenium Binding Proteins used in the matrix of the phylogenetic analysis was examined based on the information provided by previous studies (Flemetakis et al. 2002; Agalou et al. 2006; Martins Alves et al. 2019). All proteins were examined for the presence of the CC, *DEL, CxxC, HxD and HxxD motifs, which comprise the putative functional domains of the protein, documented elsewhere (Flemetakis et al. 2002; Agalou et al. 2006; Schild et al. 2014). The motifs and domains presented here were edited using Jalview (Waterhouse et al. 2009) and visualized with WebLogo 3 (http://weblogo.threeplusone.com/) (Schneider and Stephens 1990; Crooks et al. 2004).

### 3D Modeling

The X-ray structure of the hypothetical selenium-binding protein from *Sulfolobus tokodaii* ST0059 (PDB identifier: 2ece, (Yamada et al. 2008, unpublished) was obtained from PDB (Burley et al. 2021). Analysis and visualization were performed by UCSF Chimera (Pettersen et al. 2004). Topology diagrams of the 2ece coordinates were generated with PDBsum (Laskowski 2009). Annotation and highlighting of conserved residues were based on the master alignment (Data DS1, DS4).

### Sequence Alignments and Phylogenetic Trees

For phylogenetic analysis, sequences were aligned with MAFFT (Kuraku et al. 2013; Katoh et al. 2019). Alignment parameters were set to default, and the result was verified manually. Manual editing was restricted only to the N-terminus of the protein, with the constraint to include the CC motif, where possible (Data DS1).

For Bayesian inference, PartitionFinder 2 (Lanfear et al. 2012, 2016) was used to determine a phylogenetic model that best fitted the data. The best model (LG + I + G) was determined based on AICc. Four independent MCMCMC searches were performed using different random starting points (1,000,000 number of generations), with sampling every 1,000 generations. Convergence was checked visually by plotting likelihood scores vs. generation for the runs. Based on this analysis, the burn-in was set to 25%. This analysis was performed in CIPRES Science Gateway V. 3.3, a public resource for inference of large phylogenetic trees (Miller et al. 2010), visualized using iTOL (Letunic and Bork 2019) and FigTree v1.4.4 (http://tree.bio.ed.ac.uk/software/figtree/) and edited with Inkscape (Oualline and Oualline 2018).

## Results and Discussion

### Phylogenetic Profiling

*At*SBP1 was screened for homologues in more than 10,000 Uniprot proteomes with known sequences. Remarkably, only in 1,156 proteomes a SBP1 homologue was present, with 1,078 of them belonging to eukaryotic organisms. Because of some redundancy and revision issues, SBP1 homologues eventually correspond to 1,055 eukaryotic proteins. On the other hand, of the 9,117 species that lack SBP1, 1,053 are eukaryotes (Data DS2) and most of them are fungi (762), followed by 51 birds, 47 apicomplexans, 29 oomycetes and 21 kinetoplastids. Regarding the phylum of plants there are just 13 species of which 4 flowering plants with no SBP1 homologue. The potential absence of SBP homologue in those might be attributed to genome assembly or annotation issues, and not to a biological trait.

Among the detected SBP1 homologues, there are 20 proteins not characterized as selenium-binding proteins or methanethiol oxidase homologues (Upload list Data DS3 to https://www.uniprot.org/uploadlists/). This list includes some intriguing proteins, a DNA_LIGASE_A3 domain-containing protein (A0A445CL38, potentially involved in sulfur metabolism https://string-db.org/network/3818.A0A445CL38), a Rapid Alkalinization Factor (A0A1R3G161, potentially involved in cysteine and methionine metabolism and sulfur metabolism https://string-db.org/network/210143.A0A1R3G161), two *Gossypium* Adenosylhomocysteinases (A0A7J8X274, A0A7J8LSY9), a DYW_deaminase domain-containing protein (D7MH79) and a very complex Unconventional myosin-Ie (A0A3N0XSR5, http://pfam.xfam.org/protein/A0A3N0XSR5).

Adenosylhomocysteinase is a hydrolase (EC: 3.3.1.1) participating in cysteine and methionine metabolism and thus playing a crucial role in sulfur metabolism. Previous reports have indicated that bifidobacteria are unable to assimilate inorganic sulfur and consequently these bacteria need cysteine as an organic sulfur source (Hassinen et al. 1951; Ueda et al. 1983; Schell et al. 2002; Lee and O’Sullivan 2010; Ferrario et al. 2015). This requirement can be overcome by replacing cysteine with methionine. Other studies suggest that bifidobacteria could have as sulfur source sulfur-containing metabolites such as hydrogen sulfide (H_2_S), methanethiol or glutathione (Schöpping et al. 2021). Considering that SBPs are methanethiol oxidases and thus convert methanethiol to formaldehyde, H_2_S and H_2_O_2_, a modification of SBP in *Gossypium* might have occurred to achieve a more effective sulfur metabolism.

The DYW deaminase-domains are known to act in editing enzymes and are important for RNA editing and cleavage. Moreover, this domain contains the zinc-binding motif HXE and the CXXC motif, also present in SBPs and thus might be slightly misannotated.

### Structural and Functional Context of Conserved Motifs

**Fig. 1:**
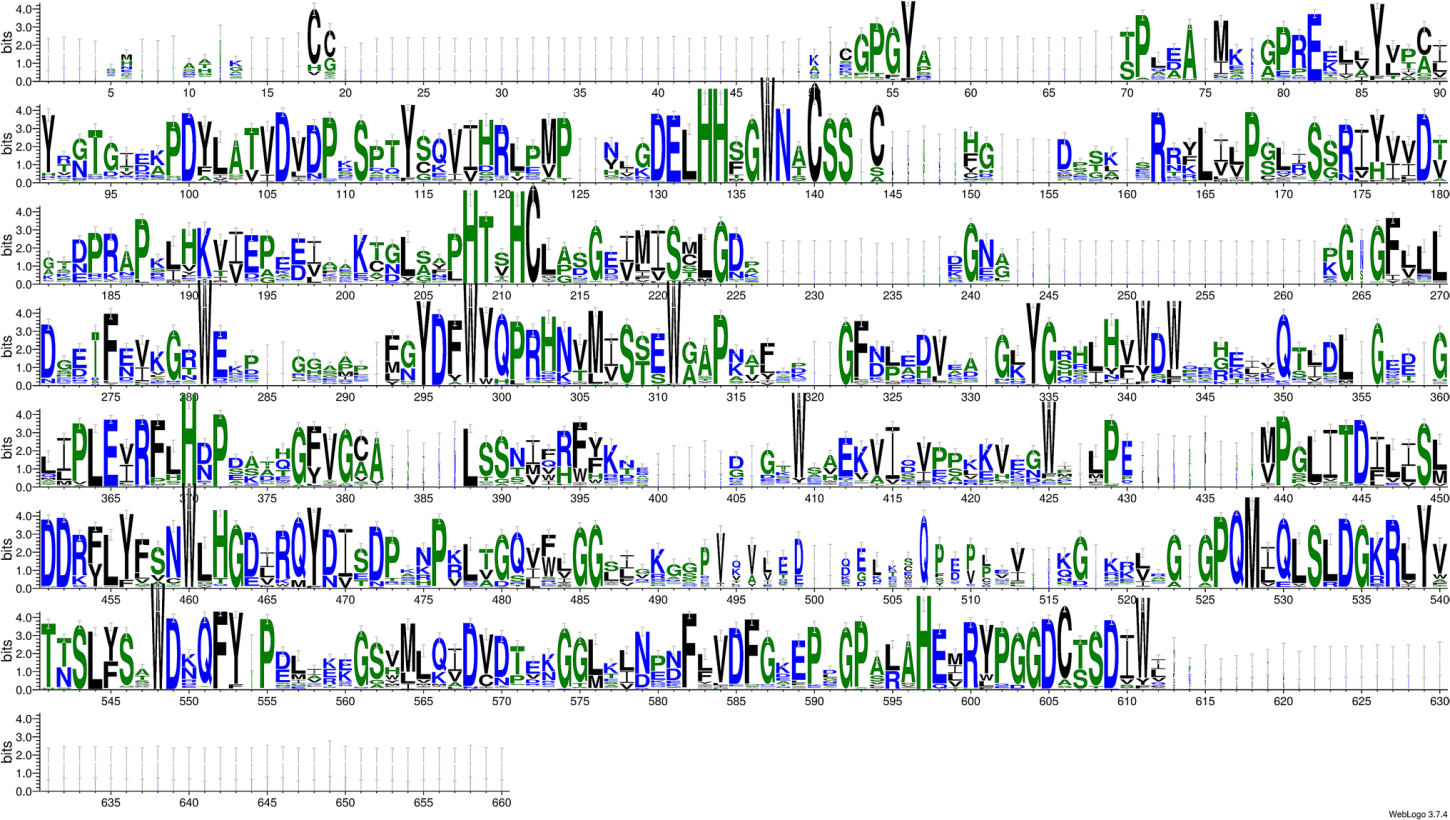
Alignment Logo. Multiple sequence alignment was done using MAFFT and after editing was upload to the online tool WebLogo **(**http://weblogo.threeplusone.com/create.cgi)

**Fig. 2:**
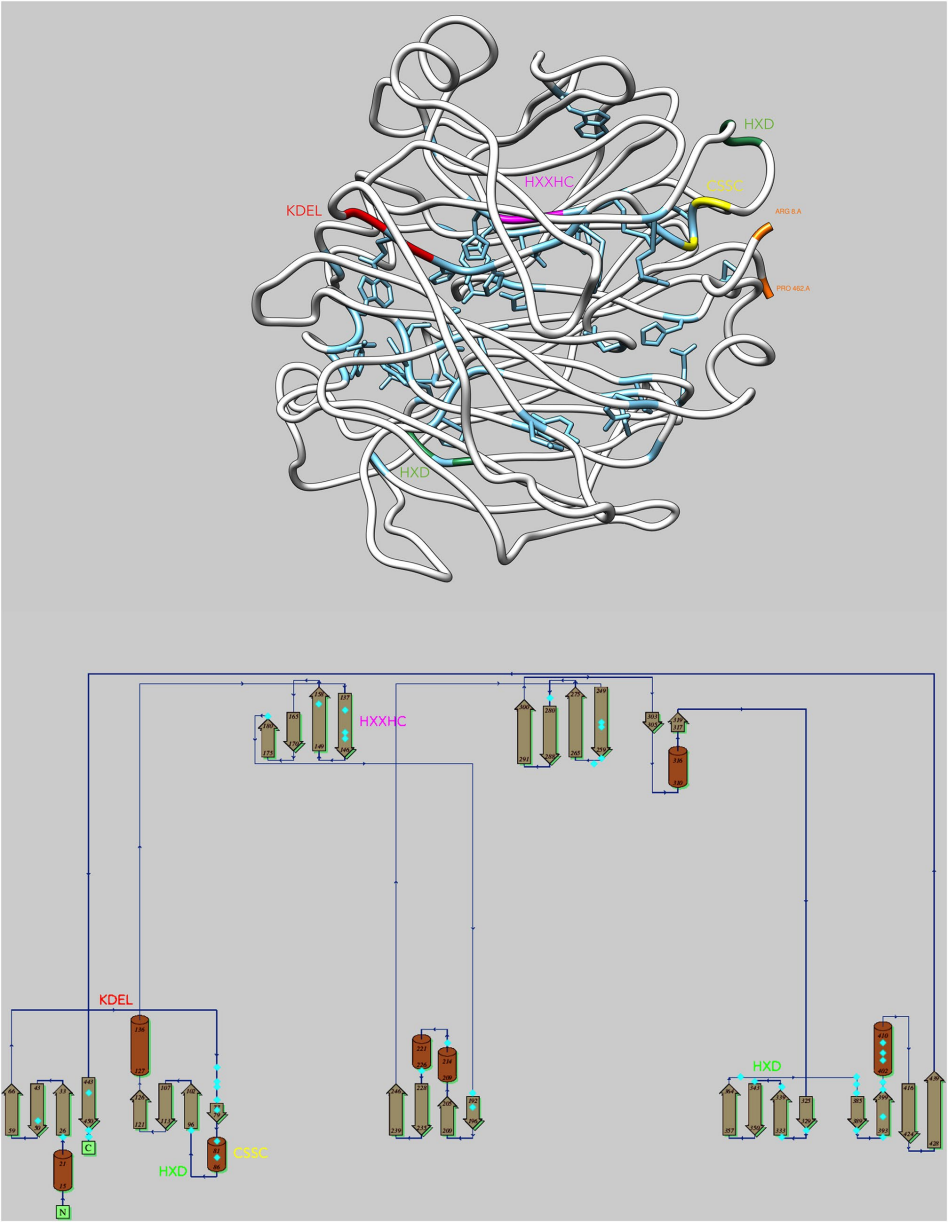
Structure of *S. tokodaii* SBP homolog (PDB cite: 2ece). Top panel: three-dimensional model for 2ece (ribbon, in gray) with key motifs shown in their structural context (KDEL in red, CSSC in yellow, HxD in green and HxxHC in magenta). The N- and C-terminal residues are also shown (top right, in orange). From the alignment of 129 representative sequences (see Methods), 43 invariant (100% identical) positions are selected, out of 454 residues available in the SBP structural model, or ~ 10% of total. Side chains of the invariant residues are also shown (main-chain and side-chain in cyan). Model generated by Chimera. Bottom panel: two-dimensional topology diagram for 2ece (strands in olive green, helices in brown). The key motifs are also shown as in the top panel. Invariant residues are approximately located (bright cyan spots) to indicate their relative position within the secondary structure elements. Residue numbers are also provided. Model generated by PDBsum

**Fig. 3:**
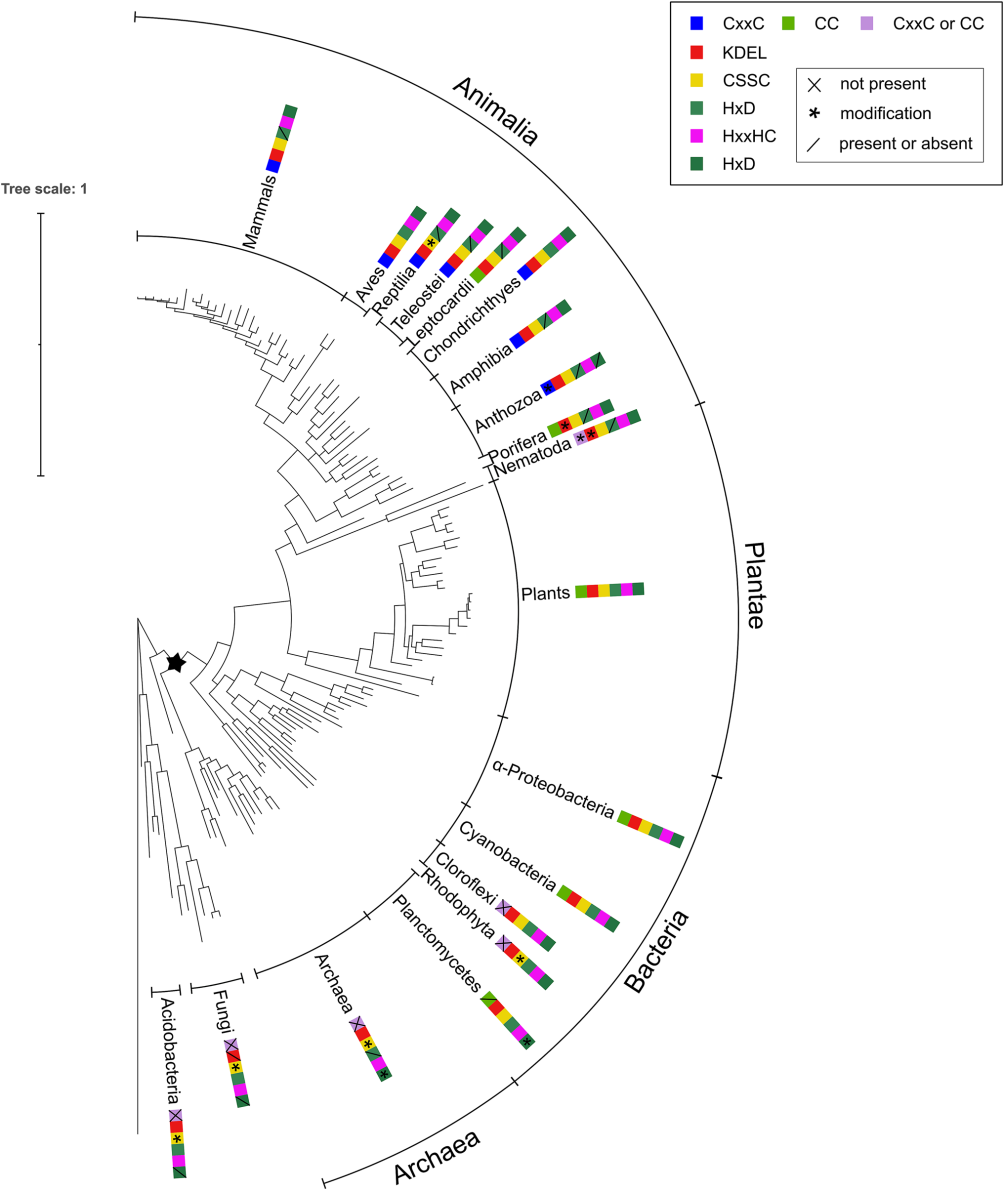
Phylogenetic relationships of SBP proteins among different species. The phylogenetic tree was generated using MrBayes software and the bacterial MTO as outgroup. iTOL was used for presentation. Sequence motifs presence is pictured by different color boxes as motifs appeared in sequences. Asterisk depicts the consistent phylogeny of Bacteria, Plants, Animals with the limited number of archaeal SBP homologs, mostly from TACK group and Halobacteriota (Euryarchaeota), as well as the anomalous distribution of homologs in a narrow range of fungal species in close association with acidobacteria

Alignment and comparison of the SBP amino acid sequences from all organisms examined in the present study, revealed the presence of conserved motifs such as CC/CXXC (Schild et al. 2014), KDEL, CSSC, HXD and HXXHC (Flemetakis et al. 2002; Agalou et al. 2006) (Fig. 1, 2). Particularly, the Se-binding motif CC in SBP1 of *Arabidopsis thaliana* (Schild et al. 2014), contains two Cys residues (Cys 21 and Cys 22) which could bind a single atom of selenium to form a R-S-Se (II)-S-R-type complex (Schild et al. 2014). The CC motif (Fig. 1, positions 18–19, Fig. 2) was found in the Plantae except for two out of three sequences of *Glycine max*, as well as in cyanobacteria and a-Proteobacteria species. Moreover, this motif was present in one from the six representatives of Anthozoa, in the representative of Porifera and Leptocardii, in one from the two Nematoda and in two out of the seven Planctomycetes (Fig. 2). This observation could be due to the fact that the CC motif has been characterized as a motif able to bind Se in photosynthetic organisms (Schild et al. 2014). In contrast, the CC motif has been substituted with CxxC (Fig. 1, 18–52, Fig. 2) in most Chordata, where it is predicted as a possible candidate for Se binding (Schild et al. 2014). On the other hand, neither the CC motif nor the CXXC motif are present in Bacteria, Archaea and Fungi (Fig. 3, S1).

The motif [G/K/R/D/N]DEL appears as *DEL (Fig. 1, positions 130–132, Fig. 2) in all examined species except *Psyscommitrella* (the only one in Bryophyta) and *Amphideon* (the only one in Sponges) which have a substitution (*DEI), while some Fungi contain *SEV (Fig. 3, Table S2). This motif is an endoplasmic reticulum (ER) retention signal of many soluble proteins contained in the cisternal lumen in eukaryotic cells (Stornaiuolo et al. 2003).

The CxxC (CSSC) motif (Fig. 1, positions 140–144, Fig. 2) appears conserved in all species except *Pelodiscus sinensis* (the only one in Testudines) which has a CSSS substitution and *Galdieria sulphuraria* (the only one in Rhodophyta) CCSS (Table S2). In the clade of Archaea (Halobacteria) it is present as CSSSC (Table S2). The CXXC motif with cysteine residues, is employed by many redox proteins for the formation, isomerization, and reduction of disulfide bonds or other redox functions. This sequence is referred to as thioredoxin-like domain (Edman et al. 1985; Kimura et al. 2004). Recent studies revealed the presence of many natural homologues of CxxC-containing proteins, in which the C-terminal Cys in the CxxC motif, is replaced with serine (the CxxS motif) (Fomenko and Gladyshev 2002). Some of these enzymes are biochemically characterized revealing an expanded repertoire of redox functions, such as the role of CxxS-containing proteins in methionine sulfoxide reduction (Kumar et al. 2002) and protein retention through transient formation of intermolecular disulfide bonds (Anelli et al. 2002). Genomic analyses determined that the CxxS motifs are highly conserved and present in structurally distinct proteins, suggesting that CxxS is a new fold-independent redox motif (Fomenko and Gladyshev 2002, 2003). Recently, it was shown that the CXXC motif of the branched-chain aminotransferase (BCAT1)–a protein which participates in myeloid leukaemia development–is crucial for the redox homeostasis and redox-mediated cellular processes having a novel antioxidant role. Particularly, CXXC motif can reduce H_2_O_2_ and utilize reducing equivalents from NADPH in this process (Hillier et al. 2022). These characteristics are consistent with the hypothesis that SBP participates in antioxidant processes and is connected with several diseases. Moreover, in silico analysis of *Theobroma cacao* SBP homologue showed interaction between CSSC motif and selenite, whereas no interaction was found with selenate (Martins Alves et al. 2019).

In addition, the putative metal binding motif with histidine residues HxD appears in two different parts of the SBP protein sequence (Fig. 1, positions 150–156 and 462–464, Fig. 2), while altered in some representative species. Particularly, the HXD in positions 150–156 of the alignment was observed only in α-Proteobacteria, Cyanobacteria, Chloroflexi, Aves and Chondrichthyes and most Plants, whereas the HXD in positions 462–464 is more conserved and absent in one Chondrichthyes, some Anthozoa, Fungi, Acidobacteria and modified in Archaea (HXE, HXN) and *Chlamydomonas reinhardtii* (RXD) (Table S2). The HxD motif is one of the highly conserved structural components of the catalytic core of protein kinases which are critical for redox signaling (Zhang et al. 2015). Furthermore, HXD motif is known as a metal-binding motif and in Tet-eleven translocation (Tet) family can bind Fe (II) and act as cofactor, as well as Ni (II) and Cd (II), with higher affinity to Ni (II) affecting the activity of Tet-mediated DNA hydroxymethylation (Yin et al. 2017).

Another highly conserved metal ion coordination motif, is the HXXH which is followed by a Cys residue creating the HXXHC motif (Fig. 1, positions 218–222, Fig. 2). This motif is present in all protein sequences used in this analysis with the exception of the outgroup (Fig. 2, Table S2). In some cases, such as the Characterising N-acetylglucosaminylphosphatidylinositol de-N-acetylase (CaGpi12) of *Candida albicans* and in the 3′-Phosphoadenosine-5′-phosphosulfate (PAPS) of human, it has been considered as important motif for the function of these enzymes by achieving binding of the substrates (Yadav et al. 2018; Zhang et al. 2022). Therefore, we hypothesize the plausible participation of this motif in the binding of SBP substrates.

Another intriguing protein trait is the presence of two clathrin-binding boxes in positions 446–451 and 582–587 (Fig. 1). The clathrin binding box consists of pLφpφp, where φ is a bulky hydrophobic amino acid and p any amino polar residue. This motif is present in clathrin binding proteins such as AP-2, AP180, amphiphysin, epsin and arrestin2 (Kang et al*.* 2009). The pLφpφp motif of SBP sequences may correlate with a previous observation of SBP linked to membrane trafficking functions (Porat et al. 2000; Agalou et al. 2006).

The above motifs that have been defined for SBP1 can be further understood within the structural context of the hypothetical selenium-binding protein of *S. tokodaii* (Fig. 2). The most conserved motifs are clustered away from the core of the molecule which is defined by a beta-propeller topology (Fig. 2). The KDEL motif is located at the end of beta strand three (residues 59–66) followed by a number of invariant positions that lead into beta strand four (positions 77–79) (Fig. 2, bottom panel). The CSSC/HxD motifs are all located prior to strand five (positions 96–102), thus not participating in the structural core of the beta propeller. Remarkably, the HxxHC motif is the only conserved motif that is located on strand eight (positions 137–146) and possesses two of the six invariant histidines (Data DS4) five of which are in close spatial proximity (positions 133, 134, 208, 211, 597 and 74, 75, 141, 144, 445, in Fig. 1 and 2ece Fig. 2, respectively). The sixth invariant histidine is located at position 370 (Fig. 1, position 260 in Fig. 2). The functional significance of this arrangement is not understood at present. In contrast to the conserved motifs mentioned above, the remaining invariant residues appear to contribute to the structural stability of the molecule, most likely performing helix-breaking (positions 214, 226) or strand-forming (positions 365–401) roles (Fig. 2, bottom panel).

### Vertical and Potential Horizontal Inheritance Across the Tree of Life

To explore the evolutionary relationship among SBP proteins, a multiple sequence alignment of protein sequences from different species was performed using MAFFT and a phylogenetic tree was generated with MrBayes software using as an outgroup the methanethiol oxidase of *Hyphomicrobium sp* (Fig. 3, Fig. S1). The identity levels between the query sequence from *Arabidopsis thaliana* and all reported homologs as well as their lengths in amino acid residues are shown as frequency distribution diagrams (Fig. S2), with average values for sequence identity 58.2% and length of 479 residues. All the members of the SBP family were divided into four major clades Archaea (Halobacteria), Bacteria (Proteobacteria, Chloroflexi, Cyanobacteria, Planctomycetes), Plants, Animalia (Mammalia, Reptilia, Amphibia, Aves, Telesostei, Leptocardii, Chondrichthyes, Porifera, Nematoda, Anthozoa). Interestingly, SBP proteins with similar domain architecture were clustered in the same clades. Moreover, the architecture of the SBP tree seems to follow the evolutionary pathway from Archaea to Protista and Bacteria, then to Plants and finally to Animalia. In more detail, there is a clade at the base of the tree with Fungi and Acidobacteria and a member of Archaea (*Sulfurisphaera tokadai*)*.*

The atypical cluster of Fungi and acidobacteria might represent a case of lateral gene transfer (LGT) between these two phyla. Fungal SBP homologs were found only in Ascomycota and in close association with acidobacteria exhibiting high similarity (identity ~ 55%). Apart from the species of Ascomycota presented in the phylogenetic tree (Fig. 3), SBP homologs were detected only in *Cladophialophora* sp., *Neofusicoccum parvum* and *Fusarium decemcellulare*, also members of Ascomycota. Acidobacteria and Ascomycota are known to form microbial communities in the soil and participate in carbon and nitrogen cycles (Challacombe et al. 2019; Kalam et al. 2020). Moreover, recent observations suggest that members of both taxa are abundant in contaminated acidic soils (Liu et al. 2022) suggesting a coordinated biochemistry. Thus, the co-occurrence of Acidobacteria and Ascomycota could have led to LGT that explains the presence of SBP homologs in the fungi.

The group of Archaea is well based and it is followed by a group of the three out of seven Planctomycetes as a base for all the other clades. The other four Planctomycetes are grouped with the other Bacteria and as a base to this clade which is comprised of α-Proteobacteria, Cyanobacteria, Chlroroflexi and Rhodophyta (Protista). The clade of Plants is following with *Chlamydomonas reinhardtii* as the base and the clade of Animals with Nematoda as base. All the Bayesian bootstrap values ranged from 54–100% (Fig. S1)**.**

All the members of Archaea (TACK group and Halobacteriota) clustered reliably, except *Sulfurisphaera tokadaii* (*Sulfolobus tokodaii*) which is grouped with the mixed clade of fungi and acidobacteria. We note an absence of detectable SBP homologs in the DPANN group and well-established methanogenic clades of Archaea, such as Methanococcales and Methanosarcinales. The relative abundance of SBP homologs in certain clades of Archaea with the aforementioned exceptions implies a potential LGT or even a massive gene loss of SBP homologs, difficult to distinguish at such scale for deep phylogenies.

Protista appear between different groups of Bacteria (Chloroflexi and Planctomycetes) and are represented by one member of Rhodophyta (sp. *Galdieria*). Bacteria are divided into four well supported groups except *Τhermobaculum terrenum* which was clustered away from the Bacteria lineage and Acidobacteria which is grouped with Fungi. The four groups are Chloroflexi with three members *Ktedonosporobacter rubrisoli*, *Dictyobacter formicarum*and and *Reticulibacter mediterranei,* Cyanobacteria with five members, α-Proteobacteria with ten representatives and Planctomycetes with seven.

The largest clade of the phylogenetic tree was divided in two large groups, the one group included the representatives of Plants and the other group the members of Animalia. In Plants, *Chlamydomonas reinhardtii* (Chlorophyta) is located at the base of this clade followed by *Physcomitrium patens* (Bryophyta) and *Selaginella moellendorffii* (Lycopodiopsida). In Animalia six groups are formed: One group with Mammals, Aves and Reptilia, second group with Teleostei, Leptocardii and Chondrichthyes, third group comprised by Amphibia, fourth with Anthozoa, fifth with Porifera and sixth with Nematoda.

The phylogenetic tree also indicates the differentiation and evolution of the characteristic SBP motifs. In more detail, the HxxHC is conserved in all representative sequences used, whereas the *DEL motif is always present except for fungi. Archaea is in the base of Bacteria, Plantae and Animalia while Acidobacteria and Fungi are basal to Archaea. The second HxD metal motif is absent from Fungi and Acidobacteria and appears with modifications in Archaea and in Planctomycetes, so the formation of this motif took place in Archaea and Planctomycetes and was maintained in the other phylogenetic groups. Likewise, evolutionary events also concern the CSSC motif where the basal clades (Acidobacteria, Fungi and Archaea) carry modifications. Moreover, the CC motif is harbored by some bacteria (α-Proteobacteria and Cyanobacteria) and remains conserved in Plants, whereas modified to CxxC in Animals. Thus, the characteristic sequence motifs of SBPs mainly appeared in Archaea and Bacteria and retained in Animals and Plants.

It is worth pointing out that out of the four major clades reported herein, the two eukaryotic taxa of animals and plants as well as the bacterial domain exhibit a phylogenetically consistent distribution pattern for SBP homologs. In contrast, the fourth category that includes a section of Archaea alongside some Fungi and Acidobacteria indicates an anomalous phylogenetic history that might include lateral gene transfer events and/or losses. This pattern (Fig. 2) is a structural perspective based on sequence phylogenies and does not reflect any known functional diversification of SBPs, as a precise molecular function is limited to just a few instances, such as *A. thaliana*, *C. elegans* and *H. sapiens.* The *C. reinhardtii* SBP homolog was recently characterized as a novel stress sensor (Koletti et al. 2022), while the human homolog plays crucial role in cancer development (Bansal et al. 1990; Xia et al. 2011; Huang et al. 2012; Ansong et al. 2015). However, the only known function is based on bacterial SBP56 which is characterized as a Cu-dependent methanethiol oxidase (MTO) (Eyice et al. 2018).

In conclusion, the exact role of Selenium Binding Proteins remains an open question, yet our analysis demonstrates their emergence from bacteria and most likely as methanethiol oxidases. The similarity degree of SBPs is high and the level of conservation is comparable to other protein families like histones, actin, ubiquitin, γ-tubulin (49%), Heat Shock (44–75%), 14-3-3 proteins and elF4E (Flemetakis et al. 2002; Agalou et al. 2006). Interestingly, the absence of SBPs in yeast from our phylogenetic profiling analysis correlates with a study where another redox related protein, the Quiescin Sulfhydryl Oxidase (QSOX), is also absent from fungi, indicating a common diversified evolutionary pathway and differentiation in time of redox response pathways in this taxon (Limor-Waisberg et al. 2013).

## Supplementary Information

Below is the link to the electronic supplementary material.

Supplementary file1 (PDF 1165 kb)

## Data Availability

Sequence, structure and taxonomic information presented in this study (Data DS1-DS5) is available on FigShare at https://doi.org/10.6084/m9.figshare.22188193 to ensure reproducibility. Additional supplementary material is available on the journal website.
