# Supplementary material for: Evolutionary Aspects of Selenium Binding Protein (SBP)
Source: J Mol Evol. 2023 Apr 11;91(4):471–81. doi: 10.1007/s00239-023-10105-4 (PMC10277263; doi:10.1007/s00239-023-10105-4)

Evolutionary aspects of Selenium Binding Protein (SBP)

Irene Dervisi[1], Chrysanthi Valassakis[1], Katerina Koletti[2], Vassilis N. Kouvelis[3], Emmanouil Flemetakis[2], Christos A. Ouzounis[4,5] and Andreas Roussis[1]

[1]Section of Botany, Department of Biology, National & Kapodistrian University of Athens, 15701 Athens, Greece

[2]Department of Biotechnology, School of Applied Biology and Biotechnology, Agricultural University of Athens, 11855, Athens, Greece.

[3]Section of Genetics and Biotechnology, Department of Biology, National & Kapodistrian University of Athens, 15701 Athens, Greece

[4]Biological Computation & Process Laboratory, Chemical Process & Energy Resources Institute, Centre for Research & Technology Hellas, Thessalonica GR-54124, Greece

[5]Biological Computation & Computational Biology Group, AIIA Lab, School of Informatics, Aristotle University of Thessalonica, Thessalonica GR-57001, Greece

Corresponding author. Email: aroussis@biol.uoa.gr

Table S1: Selenium binding proteins representatives used in the phylogenetic analysis categorized by Species, Accession Number, Query Identity (%), Protein Length (aa) and Classification.

Species	Accesion No.	Identity (%)	Protein Length (aa)	Kingdom	Phylum	Class	Order	Family
Gorilla gorilla gorilla	XP_004026685.1	61.064	514	Animalia	Chordata	Mammalia	Primates	Hominidae
Pongo abelii	XP_009242677.1	61.064	514	Animalia	Chordata	Mammalia	Primates	
Nomascus leucogenys	XP_030680407.1	60.851	514	Animalia	Chordata	Mammalia	Primates	Hylobatidae
Homo sapiens	NP_003935.2	61.064	472	Animalia	Chordata	Mammalia	Primates	Hominidae
Macaca mulatta	XP_015007568.1	61.064	472	Animalia	Chordata	Mammalia	Primates	Cercopithecidae
Callithrix jacchus	XP_002759958.1	60.213	472	Animalia	Chordata	Mammalia	Primates	Callitrichidae
Rhinolophus ferrumequinum	XP_032949704.1	61.277	472	Animalia	Chordata	Mammalia	Chiroptera	Rhinolophidae
Mustela putorius furo	XP_004776779.1	59.574	472	Animalia	Chordata	Mammalia	Carnivora	Mustelidae
Myotis lucifugus	XP_023615033.1	61.064	477	Animalia	Chordata	Mammalia	Chiroptera	Vespertilionidae
Ailuropoda melanoleuca	XP_034510167.1	59.787	472	Animalia	Chordata	Mammalia	Carnivora	Ursidae
Canis lupus familiaris	XP_005630863.2	60.000	502	Animalia	Chordata	Mammalia	Carnivora	Canidae

Felis catus	XP_019693399.1	60.213	500	Animalia	Chordata	Mammalia	Carnivora	Felidae
Loxodonta africana	XP_010587740.1	62.128	472	Animalia	Chordata	Mammalia	Proboscidea	Elephantidae
Oryctolagus cuniculus	XP_008262672.1	60.000	472	Animalia	Chordata	Mammalia	Lagomorpha	Leporidae
Equus caballus	XP_023496950.1	61.064	472	Animalia	Chordata	Mammalia	Perissodactyla	Equidae
Bos taurus	NP_001039513.1	60.000	472	Animalia	Chordata	Mammalia	Artiodactyla	Bovidae
Sus scrofa	XP_020945522.1	59.044	506	Animalia	Chordata	Mammalia	Artiodactyla	Suidae
Ictidomys tridecemlineatus	XP_005331271.1	61.489	472	Animalia	Chordata	Mammalia	Rodentia	Sciuridae
Cavia porcellus	XP_003478942.2	61.373	471	Animalia	Chordata	Mammalia	Rodentia	Caviidae
Heterocephalus glaber	XP_004854049.1	59.362	471	Animalia	Chordata	Mammalia	Rodentia	Bathyergidae
Mus musculus	NP_033176.2	60.213	472	Animalia	Chordata	Mammalia	Rodentia	Muridae
Rattus norvegicus	NP_001316822.1	61.702	472	Animalia	Chordata	Mammalia	Rodentia	
Monodelphis domestica	XP_007485480.1	59.787	471	Animalia	Chordata	Mammalia	Didelphimorphia	Didelphidae
Sarcophilus harrisii	XP_031823380.1	60.127	511	Animalia	Chordata	Mammalia	Dasyuromorphia	Dasyuridae
Pelodiscus sinensis	XP_006114005.1	55.745	472	Animalia	Chordota	Reptilia	Testudines	
Gallus gallus	NP_001264739.1	58.723	471	Animalia	Chordata	Aves	Galliformes	Phasianidae
Taeniopygia guttata	XP_041577115.1	60.000	509	Animalia	Chordata	Aves	Passeriformes	Estrildidae
Molothrus ater	XP_036254012.1	60.169	472	Animalia	Chordata	Aves	Passeriformes	Icteridae
Oryzias latipes	XP_023820002.1	60.638	472	Animalia	Chordata	Teleostei	Beloniformes	Adrianichthyidae
Oreochromis niloticus	XP_019220298.1	57.637	484	Animalia	Chordata	Teleostei	Perciformes	Cichlidae
Salmo salar	XP_014006033.2	58.936	473	Animalia	Chordata	Teleostei	Salmoniformes	Salmonidae
Danio rerio	XP_005158264.1	58.599	473	Animalia	Chordata	Teleostei	Cypriniformes	Cyprinidae
Branchiostoma floridae	XP_035657836.1	56.364	493	Animalia	Chordata	Leptocardii	Amphioxiformes	Branchiostomatidae
Scyliorhinus canicula	XP_038643176.1	60.426	517	Animalia	Chordata	Chondrichthyes	Carcharhiniformes	Scyliorhinidae
Carcharodon carcharias	XP_041037600.1	59.574	472	Animalia	Chordata	Chondrichthyes	Lamniformes	Lamnidae
Amblyraja radiata	XP_032871739.1	58.298	473	Animalia	Chordata	Chondrichthyes	Rajiformes	Rajidae
Callorhinchus milii	NP_001279677.1	59.787	472	Animalia	Chordata	Chondrichthyes	Chimaeroiformes	Callorhinchidae
Xenopus tropicalis	NP_001016750.1	61.064	472	Animalia	Chordata	Amphibia	Anura	Pipidae
Xenopus laevis	NP_001089396.1	61.489	472	Animalia	Chordata	Amphibia	Anura	Pipidae
Rana temporaria	XP_040189352.1	58.936	472	Animalia	Chordata	Amphibia	Anura	Ranidae
Bufo gargarizans	XP_044131996.1	59.236	473	Animalia	Chordata	Amphibia	Anura	Bufonidae
Acropora millepora	XP_029189081.2	61.684	472	Animalia	Cnidaria	Anthozoa	Scleractinia	Scleractinia
Pocillopora damicornis	XP_027046902.1	59.667	474	Animalia	Cnidaria	Anthozoa	Scleractinia	Pocilloporidae

Stylophora pistillata	XP_022797692.1	59.875	474	Animalia	Cnidaria	Anthozoa	Scleractinia	Pocilloporidae
Nematostella vectensis	XP_032238128.1	58.051	484	Animalia	Cnidaria	Anthozoa	Actiniaria	Edwardsiidae
Actinia tenebrosa	XP_031566459.1	58.475	480	Animalia	Cnidaria	Anthozoa	Actiniaria	Actiniidae
Exaiptasia diaphana	XP_020898889.1	57.203	480	Animalia	Cnidaria	Anthozoa	Actiniaria	Aiptasiidae
Amphimedon queenslandica	XP_019849180.1	60.169	475	Animalia	Porifera	Demospongiae	Haplosclerida	Niphatidae
Caenorhabditis elegans	NP_001255777.1	48.408	471	Animalia	Nematoda	Chromadorea	Rhabditida	Rhabditidae
Strongyloides ratti	XP_024502420.1	44.075	474	Animalia	Nematoda	Chromadorea	Rhabditida	Strongyloididae
Arabidopsis thaliana	NP_001190723.1	100.000	490	Plantae	Streptophyta	Magnoliopsida	Brassicales	Brassicaceae
Eutrema salsugineum	XP_006414821.1	92.653	487	Plantae	Streptophyta	Magnoliopsida	Brassicales	Brassicaceae
Arabidopsis thaliana	NP_193140.1	88.776	487	Plantae	Streptophyta	Magnoliopsida	Brassicales	Brassicaceae
Eutrema salsugineum	XP_006414820.1	87.347	487	Plantae	Streptophyta	Magnoliopsida	Brassicales	Brassicaceae
Arabidopsis thaliana	NP_189022.1	80.000	480	Plantae	Streptophyta	Magnoliopsida	Brassicales	Brassicaceae
Eutrema salsugineum	XP_006418837.1	78.333	478	Plantae	Streptophyta	Magnoliopsida	Brassicales	Brassicaceae
Medicago truncatula	XP_003601694.1	79.668	488	Plantae	Streptophyta	Magnoliopsida	Fabales	Fabaceae
Glycine max	XP_003552370.1	80.084	484	Plantae	Streptophyta	Magnoliopsida	Fabales	Fabaceae
Lotus japonicus	Q93VA1	79.873	487	Plantae	Streptophyta	Magnoliopsida	Malpighiales	Euphorbiaceae
Glycine max	XP_003531213.1	78.038	484	Plantae	Streptophyta	Magnoliopsida	Fabales	Fabaceae
Glycine max	XP_003524921.1	75.975	488	Plantae	Streptophyta	Magnoliopsida	Fabales	Fabaceae
Prunus persica	XP_007221280.1	78.659	491	Plantae	Tracheophyta	Magnoliopsida	Rosales	Rosaceae
Ricinus communis	XP_002520613.1	79.574	475	Plantae	Tracheophyta	Magnoliopsida	Malpighiales	Euphorbiaceae
Punica granatum	XP_031405541.1	77.917	479	Plantae	Tracheophyta	Magnoliopsida	Myrtales	Lythraceae
Hordeum vulgare	KAE8800473.1	75.751	498	Plantae	Streptophyta	Liliopsida	Poales	Poaceae
Triticum aestivum	KAF7025350.1	74.895	494	Plantae	Tracheophyta	Liliopsida	Poales	Poaceae
Triticum aestivum	KAF7039369.1	74.788	494	Plantae	Tracheophyta	Liliopsida	Poales	Poaceae
Triticum aestivum	KAF7032295.1	74.686	494	Plantae	Tracheophyta	Liliopsida	Poales	Poaceae
Oryza sativa subsp. japonica	XP_015621286.1	76.445	482	Plantae	Tracheophyta	Liliopsida	Poales	Poaceae
Zea mays	NP_001131338.2	76.000	493	Plantae	Tracheophyta	Liliopsida	Poales	Poaceae

Theobroma cacao	A0A061EKF5	80.632	476	Plantae	Tracheophyta	Magnoliopsida	Malvales	Malvaceae
Citrus clementina	XP_006421033.1	77.143	487	Plantae	Tracheophyta	Magnoliopsida	Sapindales	Rutaceae
Selaginella moellendorffii	XP_002987612.1	71.338	477	Plantae	Tracheophyta	Lycopodiopsida	Selaginellales	Selaginellaceae
Selaginella moellendorffii	XP_002983337.2	70.913	570	Plantae	Tracheophyta	Lycopodiopsida	Selaginellales	Selaginellaceae
Physcomitrium patens	XP_024388851.1	71.548	495	Plantae	Bryophyta	Bryopsida	Funariales	Funariaceae
Chlamydomonas reinhardtii	XP_001703358.1	55.625	477	Plantae	Chlorophyta	Chlorophyceae	Volvocales	Chlamydomonadace
Geminicoccus roseus	WP_027132785.1	56.383	457	Bacteria	Proteobacteria	Alphaproteobacteria	Geminoccales	Geminicoccaceae
Aurantimonas coralicida	WP_228241075.1	54.255	454	Bacteria	Proteobacteria	Alphaproteobacteria	Rhizobias	Phizobiaceae
Paracoccus seriniphilus	WP_089345033.1	53.125	454	Bacteria	Proteobacteria	Alphaproteobacteria	Rhodobacterales	Rhodobacteraceae
Sulfitobacter brevis	WP_093924074.1	54.043	454	Bacteria	Proteobacteria	Alphaproteobacteria	Rhodobacterales	Rhodobacteraceae
Pseudaestuariivita atlantica	WP_050531472.1	53.191	449	Bacteria	Proteobacteria	Alphaproteobacteria		Rhodobacteraceae
Mesorhizobium amorphae	WP_006201402.1	52.766	451	Bacteria	Proteobacteria	Alphaproteobacteria	Rhizobias	Phizobiaceae
Candidatus Rhodobacter lobularis	WP_049644243.1	53.830	448	Bacteria				
Ruegeria pomeroyi	WP_011048093.1	53.830	449	Bacteria	Proteobacteria	Alphaproteobacteria	Rhodobacterales	Rhodobacteraceae
Thalassobium sp. R2A62	WP_009158083.1	52.340	452	Bacteria				
Octadecabacter arcticus	WP_015494695.1	52.229	452	Bacteria	Proteobacteria	Alphaproteobacteria	Rhodobacterales	Rhodobacteraceae
Tolypothrix bouteillei	WP_038086674.1	58.351	450	Bacteria	Cyanobacteria	Cyanobacterila	Cyanobacteriales	Nostocaceae
Microseira wollei	WP_226587261.1	57.928	450	Bacteria	Cyanobacteria	Cyanobacterila	Cyanobacteriales	Oscillatoriaceae
Hassallia byssoidea	WP_039740169.1	57.505	450	Bacteria	Cyanobacteria	Cyanobacterila	Cyanobacteriales	Nostocaceae
Chroococcidiopsis cubana	WP_106168649.1	57.872	451	Bacteria	Cyanobacteria	Cyanobacterila	Cyanobacteriales	Chroococcidiopsidac
Gloeocapsopsis dulcis	WP_105219090.1	58.774	457	Bacteria	Cyanobacteria	Cyanobacterila	Cyanobacteriales	Chroococcidiopsidac
Ktedonosporobacter rubrisoli	WP_129889517.1	56.838	456	Bacteria	Chloroflexi	Ktenobacteria	Ktenobacterales	Ktenobacteraceae
Dictyobacter formicarum	WP_201362033.1	56.559	453	Bacteria	Chloroflexi	Ktenobacteria	Ktenobacterales	Ktenobacteraceae
Reticulibacter mediterranei	WP_220210928.1	55.484	459	Bacteria	Chloroflexi	Ktenobacteria	Ktenobacterales	Ktenobacteraceae
Galdieria sulphuraria	XP_005706028.1	52.155	451	Plantae	Rhodophyta	Cyanidiophyceae	Cyanidiales	Galdieriaceae

Mariniblastus fucicola	WP_084417348.1	52.866	478	Bacteria	Planctomycetota	Planctomycetes	Pirellulales	Pirellulaceae
Lignipirellula cremea	WP_145056384.1	51.919	480	Bacteria	Planctomycetota	Planctomycetes	Pirellulales	Pirellulaceae
Bythopirellula goksoyrii	WP_210417703.1	50.847	481	Bacteria	Planctomycetota	Planctomycetes	Pirellulales	Pirellulaceae
Bythopirellula polymerisocia	WP_146447520.1	49.372	476	Bacteria	Planctomycetota	Planctomycetes	Pirellulales	Pirellulaceae
Gemmata massiliana	WP_162666735.1	54.968	473	Bacteria	Planctomycetota	Planctomycetes	Gemmatales	Gemmataceae
Gemmata palustris	WP_210660059.1	54.184	475	Bacteria	Planctomycetota	Planctomycetes	Gemmatales	Gemmataceae
Thermogemmata fonticola	WP_194536478.1	49.478	493	Bacteria	Planctomycetota	Planctomycetes	Gemmatales	Gemmataceae
Haloferax mediterranei	WP_004056264.1	46.352	464	Archaea	Euryarchaeota	Halobacteria	Halobacteriales	Haloferacaceae
Halogeometricum borinquense	WP_006057024.1	45.161	463	Archaea	Euryarchaeota	Halobacteria	Halobacteriales	Halobacteriaceae
Natronorubrum sulfidifaciens	WP_008162880.1	46.022	467	Archaea	Euryarchaeota	Halobacteria	Halobacteriales	Halobacteriaceae
Natronococcus amylolyticus	WP_005555130.1	48.712	466	Archaea	Euryarchaeota	Halobacteria	Halobacteriales	Halobacteriaceae
Natronorubrum bangense	WP_006065239.1	47.845	467	Archaea	Euryarchaeota	Halobacteria	Halobacteriales	Halobacteriaceae
Haloterrigena turkmenica	WP_012944756.1	47.537	465	Archaea	Euryarchaeota	Halobacteria	Halobacteriales	Halobacteriaceae
Haloarcula marismortui	WP_011224072.1	48.498	463	Archaea	Euryarchaeota	Halobacteria	Halobacteriales	Halobacteriaceae
Haloterrigena turkmenica	WP_012945293.1	48.932	462	Archaea	Euryarchaeota	Halobacteria	Halobacteriales	Halobacteriaceae
Haladaptatus paucihalophilus	WP_018128659.1	49.571	466	Archaea	Euryarchaeota	Halobacteria	Halobacteriales	Halobacteriaceae
Halococcus hamelinensis	WP_007693083.1	48.333	463	Archaea	Euryarchaeota	Halobacteria	Halobacteriales	Halobacteriaceae
Halogranum salarium	WP_009374922.1	47.537	472	Archaea	Euryarchaeota	Halobacteria	Halobacteriales	Halobacteriaceae
Natronococcus amylolyticus	WP_049892000.1	45.203	469	Archaea	Euryarchaeota	Halobacteria	Halobacteriales	Halobacteriaceae
Halorubrum aidingense	WP_007997729.1	46.581	466	Archaea	Euryarchaeota	Halobacteria	Halobacteriales	Halobacteriaceae
Natronorubrum bangense	WP_006065829.1	46.581	466	Archaea	Euryarchaeota	Halobacteria	Halobacteriales	Halobacteriaceae
Thermobaculum terrenum	WP_012875254.1	48.380	446	Bacteria	Chloroflexi	Chloroflexia	Thermomicrobiales	Thermomicrobiacea
Sulfurisphaera tokodaii	WP_010977998.1	37.632	457	Archaea	Thermoproteota	Thermoproteia	Sulfolobales	Sulfolobaceae
Edaphobacter modestus	WP_130420317.1	38.397	457	Bacteria	Acidobacteria		Acidobacteriales	Acidobacteriaceae
Luteitalea pratensis	WP_110168895.1	36.952	462	Bacteria	Acidobacteria	Vicinamibacteria		Vicinamibacteracea
Granulicella sp. dw_53	WP_213804695.1	38.316	457	Bacteria	Acidobacteria		Acidobacteriales	Acidobacteriaceae

Lasiodiplodia theobromae	XP_035371643.1	36.122	470	Fungi	Ascomycota	Dothideomycetes	Botryosphaeriales	Botryosphaeriaceae
Diplodia corticola	XP_020132237.1	33.398	563	Fungi	Ascomycota	Dothideomycetes	Botryosphaeriales	Botryosphaeriaceae
Rhinocladiella mackenziei CBS 650.93	XP_013272585.1	35.908	459	Fungi	Ascomycota	Eurotiomycetes	Chaetothyriales	Herpotrichiellaceae
Fonsecaea multimorphosa CBS 102226	XP_016629787.1	34.855	458	Fungi	Ascomycota	Eurotiomycetes	Chaetothyriales	Herpotrichiellaceae
Fonsecaea erecta	XP_018694030.1	35.983	458	Fungi	Ascomycota	Eurotiomycetes	Chaetothyriales	Herpotrichiellaceae
Exophiala aquamarina CBS 119918	XP_013254020.1	35.095	460	Fungi	Ascomycota	Eurotiomycetes	Chaetothyriales	Herpotrichiellaceae
Kocuria sp. cx-455	WP_190807968.1	35.685	462	Bacteria	Acidobacteria		Acidobacteriales	Micrococcaceae
Hyphomicrobium sp	A0A291P0C1.1	23.400	416	Bacteria	Proteobacteria	Alphaproteobacteria	Rhizobias	

Table S2: Presence of SBPs characteristic motifs of the representatives used for the construction of the phylogenetic tree.

Accession No.	Species	CxxC	CC	GDEL	HH	GWN	CSSC	HxD	HxxHC	W	HxD	C
XP_004026685.1	*Gorilla gorilla gorilla*	+	-	KDEL	+	+	+	-	+	+	+	+
XP_009242677.1	*Pongo abelii*	+	-	KDEL	+	+	+	-	+	+	+	+
XP_030680407.1	*Nomascus leucogenys*	+	-	KDEL	+	+	+	-	+	+	+	+
NP_003935.2	*Homo sapiens*	+	-	KDEL	+	+	+	-	+	+	+	+
XP_015007568.1	*Macaca mulatta*	+	-	KDEL	+	+	+	-	+	+	+	+
XP_002759958.1	*Callithrix jacchus*	+	-	KDEL	+	+	+	-	+	+	+	+
XP_032949704.1	*Rhinolophus ferrumequinum*	+	-	KDEL	+	+	+	-	+	+	+	+
XP_004776779.1	*Mustela putorius furo*	+	-	KDEL	+	+	+	-	+	+	+	+
XP_023615033.1	*Myotis lucifugus*	+	-	KDEL	+	+	+	-	+	+	+	+
XP_034510167.1	*Ailuropoda melanoleuca*	+	-	KDEL	+	+	+	-	+	+	+	+
XP_005630863.2	*Canis lupus familiaris*	+	-	RDEL	+	+	+	-	+	+	+	+
XP_019693399.1	*Felis catus*	+	-	KDEL	+	+	+	-	+	+	+	+
XP_010587740.1	*Loxodonta africana*	+	-	KDEL	+	+	+	-	+	+	+	+
XP_008262672.1	*Oryctolagus cuniculus*	+	-	KDEL	+	+	+	-	+	+	+	+
XP_023496950.1	*Equus caballus*	+	-	KDEL	+	+	+	-	+	+	+	+
NP_001039513.1	*Bos taurus*	+	-	KDEL	+	+	+	-	+	+	+	+
XP_020945522.1	*Sus scrofa*	+	-	KDEL	+	+	+	-	+	+	+	+
XP_005331271.1	*Ictidomys tridecemlineatus*	+	-	KDEL	+	+	+	-	+	+	+	+
XP_003478942.2	*Cavia porcellus*	-	-	KDEL	+	+	+	-	+	+	+	+
XP_004854049.1	*Heterocephalus glaber*	-	-	KDEL	+	+	+	-	+	+	+	+
NP_033176.2	*Mus musculus*	+	-	KDEL	+	+	+	-	+	+	+	+
NP_001316822.1	*Rattus norvegicus*	+	-	KDEL	+	+	+	-	+	+	+	+
XP_007485480.1	*Monodelphis domestica*	+	-	KDEL	+	+	+	-	+	+	+	+
XP_031823380.1	*Sarcophilus harrisii*	+	-	KDEL	+	+	+	-	+	+	+	+
XP_006114005.1	*Pelodiscus sinensis*	+	-	RDEL	+	+	CSSS	-	+	+	+	+
NP_001264739.1	*Gallus gallus*	+	-	KDEL	+	+	+	-	+	+	+	-
XP_041577115.1	*Taeniopygia guttata*	+	-	GDEL	+	+	+	+	+	+	+	+
XP_036254012.1	*Molothrus ater*	+	-	GDEL	+	+	+	+	+	+	+	+
XP_023820002.1	*Oryzias latipes*	+	-	RDEL	+	+	+	-	+	+	+	+
XP_019220298.1	*Oreochromis niloticus*	+	-	RDEL	+	+	+	-	+	+	+	+
XP_014006033.2	*Salmo salar*	+	-	NDEL	+	+	+	-	+	+	+	+

XP_005158264.1	*Danio rerio*	+	-	KDEL	+	+	+	-	+	+	+	+
XP_035657836.1	*Branchiostoma floridae*	-	+	KDEL	+	+	+	-	+	+	+	+
XP_038643176.1	*Scyliorhinus canicula*	+	-	NDEL	+	+	+	+	+	+	+	+
XP_041037600.1	*Carcharodon carcharias*	-	-	KDEL	+	+	+	+	+	+	-	+
XP_032871739.1	*Amblyraja radiata*	+	-	NDEL	+	+	+	-	+	+	+	+
NP_001279677.1	*Callorhinchus milii*	+	-	GDEL	+	+	+	+	+	+	+	+
NP_001016750.1	*Xenopus tropicalis*	+	-	NDEL	+	+	+	-	+	+	+	+
NP_001089396.1	*Xenopus laevis*	+	-	NDEL	+	+	+	-	+	+	+	+
XP_040189352.1	*Rana temporaria*	+	-	NDEL	+	+	+	-	+	+	+	+
XP_044131996.1	*Bufo gargarizans*	CRSSC	-	DDEL	+	+	+	-	+	+	+	+
XP_029189081.2	*Acropora millepora*	-	+	GDEL	+	+	+	-	+	+	+	+
XP_027046902.1	*Pocillopora damicornis*	CxC		GDEL	+	+	+	-	+	+	+	+
XP_022797692.1	*Stylophora pistillata*	CxC		GDEL	+	+	+	-	+	+	+	+
XP_032238128.1	*Nematostella vectensis*	CxC	-	GDEL	+	+	+	-	+	+	-	+
XP_031566459.1	*Actinia tenebrosa*	-	-	GDEL	+	+	+	-	+	+	-	+
XP_020898889.1	*Exaiptasia diaphana*	-	-	GDEL	+	+	+	-	+	+	-	+
XP_019849180.1	*Amphimedon queenslandica*	-	+	EDEI	+	+	+	-	+	+	+	+
NP_001255777.1	*Caenorhabditis elegans*	CXXXC	-	GDEV	+	+	+	-	+	+	+	+
XP_024502420.1	*Strongyloides ratti*	-	+	NDEV	+	+	+	-	+	+	+	+
NP_001190723.1	*Arabidopsis thaliana*	-	+	GDEL	+	+	+	+	+	+	+	+
XP_006414821.1	*Eutrema salsugineum*	-	+	GDEL	+	+	+	+	+	+	+	+
NP_193140.1	*Arabidopsis thaliana*	-	+	GDEL	+	+	+	+	+	+	+	+
XP_006414820.1	*Eutrema salsugineum*	-	+	GDEL	+	+	+	+	+	+	+	+
NP_189022.1	*Arabidopsis thaliana*	-	+	EDEL	+	+	+	-	+	+	+	+
XP_006418837.1	*Eutrema salsugineum*	-	+	GDEL	+	+	+	-	+	+	+	+
XP_003601694.1	*Medicago truncatula*	-	+	GDEL	+	+	+	+	+	+	+	+
XP_003552370.1	*Glycine max*	-	+	GDEL	+	+	+	+	+	+	+	+
Q93VA1	*Lotus japonicus*	-	+	GDEL	+	+	+	+	+	+	+	+
XP_003531213.1	*Glycine max*	-	-	GDEL	+	+	+	-	+	+	+	+
XP_003524921.1	*Glycine max*	-	-	GDEL	+	+	+	-	+	+	+	+
XP_007221280.1	*Prunus persica*	-	+	GDEL	+	+	+	-	+	+	+	+
XP_002520613.1	*Ricinus communis*	-	+	GDEL	+	+	+	+	+	+	+	+
XP_031405541.1	*Punica granatum*	-	+	GDEL	+	+	+	+	+	+	+	+

KAE8800473.1	*Hordeum vulgare*	-	+	GDEL	+	+	+	+	+	+	+	+
KAF7025350.1	*Triticum aestivum*	-	+	GDEL	+	+	+	+	+	+	+	+
KAF7039369.1	*Triticum aestivum*	-	+	GDEL	+	+	+	+	+	+	+	+
KAF7032295.1	*Triticum aestivum*	-	+	GDEL	+	+	+	+	+	+	+	+
XP_015621286.1	*Oryza sativa subsp. japonica*	-	+	GDEL	+	+	+	+	+	+	+	+
NP_001131338.2	*Zea mays*	-	+	GDEL	+	+	+	+	+	+	+	+
A0A061EKF5	*Theobroma cacao*	-	+	GDEL	+	+	+	+	+	+	+	+
XP_006421033.1	*Citrus clementina*	-	+	GDEL	+	+	+	-	+	+	+	+
XP_002987612.1	*Selaginella moellendorffii*	-	+	GDEL	+	+	+	-	+	+	+	+
XP_002983337.2	*Selaginella moellendorffii*	-	+	DDEL	+	+	+	-	+	+	+	+
XP_024388851.1	*Physcomitrium patens*	-	+	DDEI	+	+	+	+	+	+	+	+
XP_001703358.1	*Chlamydomonas reinhardtii*	-	+	GDEL	+	+	+	+	+	+	RGD	+
WP_027132785.1	*Geminicoccus roseus*	-	+	GDEL	+	+	+	+	+	+	+	+
WP_228241075.1	*Aurantimonas coralicida*	-	+	GDEL	+	+	+	-	+	+	+	+
WP_089345033.1	*Paracoccus seriniphilus*	-	+	GDEL	+	+	+	-	+	+	+	+
WP_093924074.1	*Sulfitobacter brevis*	-	+	GDEL	+	+	+	-	+	+	+	+
WP_050531472.1	*Pseudaestuariivita atlantica*	-	+	GDEL	+	+	+	-	+	+	+	-
WP_006201402.1	*Mesorhizobium amorphae*	-	+	GDEL	+	+	+	+	+	+	+	+
WP_049644243.1	*Candidatus Rhodobacter lobularis*	-	+	GDEL	+	+	+	+	+	+	+	+
WP_011048093.1	*Ruegeria pomeroyi*	-	+	GDEL	+	+	+	+	+	+	+	+
WP_009158083.1	*Thalassobium sp. R2A62*	-	+	GDEL	+	+	+	+	+	+	+	+
WP_015494695.1	*Octadecabacter arcticus*	-	+	GDEL	+	+	+	+	+	+	+	+
WP_038086674.1	*Tolypothrix bouteillei*		+	GDEL	+	+	+	+	+	+	+	-
WP_226587261.1	*Microseira wollei*		+	GDEL	+	+	+	+	+	+	+	+
WP_039740169.1	*Hassallia byssoidea*		+	GDEL	+	+	+	+	+	+	+	+
WP_106168649.1	*Chroococcidiopsis cubana*		+	GDEL	+	+	+	+	+	+	+	+
WP_105219090.1	*Gloeocapsopsis dulcis*		+	GDEL	+	+	+	+	+	+	+	+
WP_129889517.1	*Ktedonosporobacter rubrisoli*	-	-	GDEL	+	+	+	+	+	+	+	+
WP_201362033.1	*Dictyobacter formicarum*	-	-	GDEL	+	+	+	+	+	+	+	+

WP_220210928.1	*Reticulibacter mediterranei*	-	-	GDEL	+	+	+	-	+	+	+	+
WP_012875254.1	*Thermobaculum terrenum*	-	-	GDEL	+	-	CSSSAC	-	+	+	+	+
XP_005706028.1	*Galdieria sulphuraria*	-	-	GDEL	+	+	CCSS	-	+	+	+	+
WP_084417348.1	*Mariniblastus fucicola*	-	+	GDEL	+	+	+	-	+	+	+	+
WP_145056384.1	*Lignipirellula cremea*	-	+	GDEL	+	+	+	-	+	+	+	-
WP_210417703.1	*Bythopirellula goksoyrii*	-	-	GDEL	+	+	+	-	+	+	+	-
WP_146447520.1	*Bythopirellula polymerisocia*	-	-	GDEL	+	+	+	-	+	+	+	+
WP_162666735.1	*Gemmata massiliana*	-	-	GDEL	+	+	+	-	+	+	HGE	+
WP_210660059.1	*Gemmata palustris*	-	-	GDEL	+	+	+	-	+	+	HGE	+
WP_194536478.1	*Thermogemmata fonticola*	-	-	GDEL	+	+	+	-	+	+	HGR	+
WP_004056264.1	*Haloferax mediterranei*	-	-	GDEL	+	+	CSSSC	-	+	+	HGE	+
WP_006057024.1	*Halogeometricum borinquense*	-	-	GDEL	+	+	CSSSC	-	+	+	HGE	+
WP_008162880.1	*Natronorubrum sulfidifaciens*	-	-	GDEL	+	+	CSSSC	-	+	+	HGE	+
WP_005555130.1	*Natronococcus amylolyticus*	-	-	GDEL	+	+	CSSSC	-	+	+	HGE	+
WP_006065239.1	*Natronorubrum bangense*	-	-	GDEL	+	+	CSSSC	-	+	+	HGE	+
WP_012944756.1	*Haloterrigena turkmenica*	-	-	GDEL	+	+	CSSSC	-	+	+	HGE	+
WP_011224072.1	*Haloarcula marismortui*	-	-	GDEL	+	+	CSSSC	-	+	+	HGE	+
WP_012945293.1	*Haloterrigena turkmenica*	-	-	GDEL	+	+	CSSSC	-	+	+	-	+
WP_018128659.1	*Haladaptatus paucihalophilus*	-	-	GDEL	+	+	CSSSC	-	+	+	+	+
WP_007693083.1	*Halococcus hamelinensis*	-	-	GDEL	+	+	CSSSC	-	+	+	+	+
WP_009374922.1	*Halogranum salarium*	-	-	GDEL	+	+	CSSSC	-	+	+	+	+
WP_049892000.1	*Natronococcus amylolyticus*	-	-	GDEL	+	+	CSSSC	-	+	+	HGN	+
WP_007997729.1	*Halorubrum aidingense*	-	-	GDEL	+	+	CSSSC	-	+	+	+	+
WP_006065829.1	*Natronorubrum bangense*	-	-	GDEL	+	+	CSSSC	-	+	+	+	+
WP_010977998.1	*Sulfurisphaera tokodaii*	-	-	NDEL	+	+	CSS	-	+	+	-	+
WP_130420317.1	*Edaphobacter modestus*	-	-	GDEL	+	+	CSS	-	+	+	-	+

WP_110168895.1	*Luteitalea pratensis*	-	-	GDEL	+	+	+	-	+	+	-	+
WP_213804695.1	*Granulicella sp. dw_53*	-	-	GDEL	+	+	CSS	-	+	+	-	+
WP_190807968.1	*Kocuria sp. cx-455*	-	-	PDEF	+	+	CSS	-	+	+	-	+
XP_035371643.1	*Lasiodiplodia theobromae*	-	-	GSEV	+	+	CSS	-	+	+	-	+
XP_020132237.1	*Diplodia corticola*	-	-	GSEV	+	+	CSS	-	+	+	-	+
XP_013272585.1	*Rhinocladiella mackenziei CBS 650.93*	-	-	GDEL	+	+	CSS	-	+	+	-	+
XP_016629787.1	*Fonsecaea multimorphosa CBS 102226*	-	-	DEL	+	+	CSS	-	+	+	-	+
XP_018694030.1	*Fonsecaea erecta*	-	-	GDEL	+	+	CSS	-	+	+	-	+
XP_013254020.1	*Exophiala aquamarina CBS 119918*	-	-	GQEL	+	+	CSS	-	+	+	-	+
A0A291P0C1.1	*Hyphomicrobium sp*	-	-	-	+	-	-	-	-	+	-	-

Figure S1: The phylogenetic tree was generated using MrBayes software and the bacterial MTO as outgroup. FigTree v1.4.4 (http://tree.bio.ed.ac.uk/software/figtree/) was used for this presentation. All the Bayesian bootstrap values ranged from 54-100%.

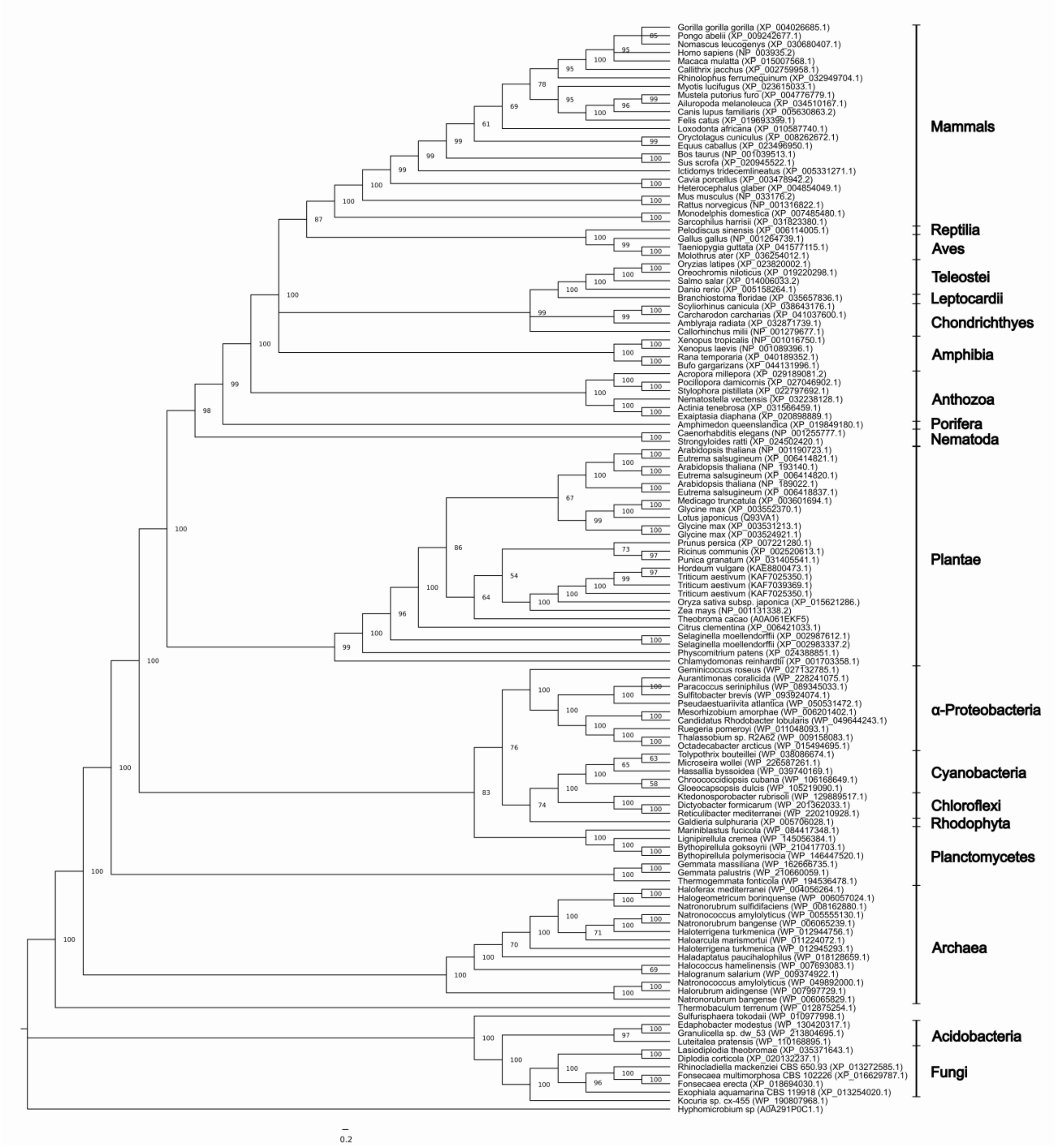

Figure S2: Frequency distributions for the reported SBP homologs: (top panel) - sequence identity to the query *A. thaliana* SBP at % level, (bottom panel) - length of protein sequence in amino acid residues.

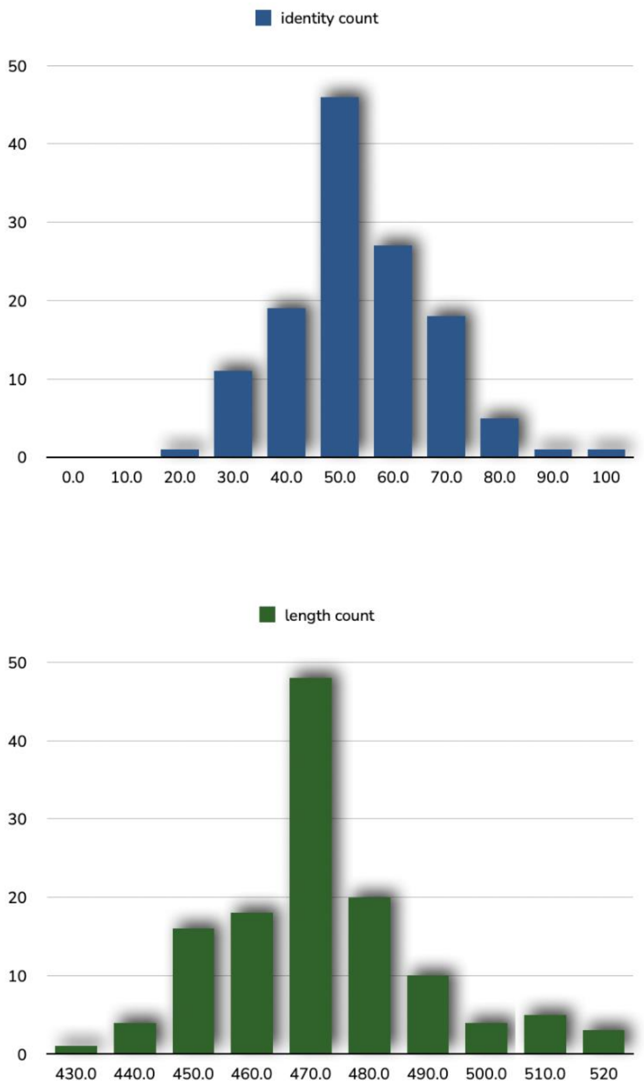

Supplement: Supplementary file 1 — Supplementary file1 (PDF 1165 kb) [file 239_2023_10105_MOESM1_ESM.pdf]
